# Direct and indirect associations of management support with compliance with laboratory safety policies: the mediating roles of perceived trust and self-efficacy

**DOI:** 10.3389/fpubh.2026.1918077

**Published:** 2026-09-23

**Authors:** Lihui Hong, Keda Yang, Jie Qu

**Affiliations:** Zhejiang Shuren University, Hangzhou, China

**Keywords:** biomedical laboratory, compliance behavior, laboratory safety policies, management support, perceived trust, self-efficacy, theory of planned behavior

## Abstract

**Background:**

Laboratory safety policies (LSP) are critical for ensuring the safety of research environments. Although studies have explored compliance with information security policies, user compliance behavior with laboratory safety policies remains under-explored.

**Methods:**

This study investigates the associations of Management Support (MS) with compliance with LSP, including direct and indirect pathways through Self-efficacy (SE) and Perceived Trust (PT), using data from 510 university biomedical laboratory users (93.72% of whom were undergraduates). Partial least squares structural equation modeling (PLS-SEM) was applied to analyse the direct and indirect associations.

**Results:**

MS showed a significant direct association with user compliance behavior (UCB) (β = 0.247, *p* = 0.003) and significant indirect associations via PT (indirect β = 0.332) and SE (indirect β = 0.198), supporting partial mediation.

**Conclusion:**

The indirect association via PT was numerically larger than that via SE (indirect β = 0.332 vs. 0.198). However, a bootstrap test of the difference between the two indirect effects (5,000 sub-samples) yielded a 95% confidence interval of [–0.117, 0.465], which includes zero, indicating that the difference was not statistically significant. Nevertheless, both indirect pathways were significant, underscoring the importance of both institutional trust and self-efficacy in promoting laboratory safety compliance.

## 1 Introduction

Accidents caused by improper operations by laboratory users account for four-fifths of the total, far exceeding those triggered by physical factors (1). No matter how good the security system is implemented in an organization, ultimately the security rating will depend on appropriate user behavior (2). Unacceptable user behavior is often referred to as human error (3), which can be defined as “a change in human performance which causes a deviation from a desired success path, which then leads to an undesired or unplanned result” (4). Many studies have found that human error is a major problem in laboratory safety (1). If an organization cannot control and manage its laboratory by implementing its laboratory safety policies, human error may pose significant safety risks (5).

Numerous studies have reported laboratory safety incidents in organizations, with over 80% of safety incidents attributable to user behavior. Despite additional resources being invested in technology-based safety solutions, the rate of safety incidents caused by user behavior continues to rise (6). Samy et al. (7) argued that human error is a major threat to the security of the healthcare industry. According to a report by Symantec Corporation (8), the healthcare sector had the largest percentage of security incidents. Laboratory safety incidents in the healthcare industry are due to a lack of safety awareness, poor safety skills, inadequate safety monitoring and enforcement, and inappropriate laboratory safety practices among users (9). Recent studies in clinical laboratory settings have similarly highlighted the importance of institutional support and safety training in shaping laboratory safety behavior (10, 11). Therefore, both government bodies and healthcare industry management should consider and invest not only in the technical aspects of safety systems, but also in human resources.

## 2 Theoretical review

### 2.1 Laboratory safety policies compliance behavior

Safety compliance behavior refers to a series of activities carried out by users in the workplace to ensure the safety of the working environment (12). An organization's laboratory safety policy typically encompasses several key areas, such as safety culture development, safety management systems, safety education and training, safety inspections and hazard identification, personal protective equipment (PPE), and equipment safety control (13). Laboratory safety compliance behaviors may influence the success or failure of laboratory safety procedures within an organization, particularly in medical and biological laboratories. Therefore, it is crucial for organizations to promote laboratory safety behaviors and regulate unacceptable laboratory safety behaviors among users (14). If users' compliance with laboratory safety protocols is acceptable, safety incidents can be minimized, and the efficiency of laboratory safety management will be improved (15).

### 2.2 Theory of planned behavior

The theory of planned behavior (TPB) has been used by previous researchers to explain user behavior in information system studies (16). It is also used to explain the indirect impact of management support on users' compliance with information security policies (17). TPB has been applied extensively to examine user acceptance of information systems, which are designed to predict human behavior (18). This theory proposed by Ajzen (19) postulates three conceptually independent determinant of intention: attitude, subjective norms (SNs) and perceived behavioral control (PBC). Ajzen (19) defined attitude as a “degree to which a person has a favorable or unfavorable evaluation or appraisal of the behavior in question” (p. 12). Meanwhile, SNs are determined by users' perceptions of other people's opinions in terms of whether or not he/she should adopt appropriate behaviors (20). Other people's opinions in this context are the opinions of superiors and colleagues. However, in this study, the researchers have only highlighted superiors' behavior because users' behavior in organizations is most often influenced by their superiors, who are also the leaders in the organization, such as director, manager and supervisor, among others. Users in organizations tend to do what they have been asked to do and to respect their superiors, which can have positive or negative effects (21). This is supported by previous findings, which have indicated that superior behavior has the greatest impact on users' information security behavior (20). Leaders should demonstrate positive security behavior and encourage users to comply with ISPs (22). Thus, research suggests that adequate security culture can be inculcated within organizations.

### 2.3 Perceived behavioral control

From the perspective of TPB, PBC can be described as a user's perceptions of his or her own ability (23), which can be heightened through education and training provided by management. According to Taylor and Todd (24), the PBC construct has two components: self-efficacy (SE) and facilitating conditions.

#### 2.3.1 Self-efficacy

Kolil et al. (25) posits that students' comprehension of laboratory safety and their ability to comply with safety protocols constitute an integral component of their self-efficacy. It can be developed through the ongoing acquisition of knowledge (26). The SE construct originates from social cognitive theory (27) and determines how people feel, think and what motivates them to behave in certain ways, based on cognitive, motivational, affective, social influence and selection processes (28). The notion that SE can be enhanced through training programs provided by management has been supported by researchers (29). Self-efficacy (SE) in laboratory safety compliance encompasses not only the proper use of safety equipment but also safety precautions related to the handling of equipment and materials (including reagents and laboratory animals).

#### 2.3.2 Facilitating conditions

Facilitating conditions, the second component of PBC, relate to facilities needed to ensure that users engage in behaviors required of them by the organization (e.g. money, time, and specialized resources) (24). Education and training programs can enhance users' laboratory safety awareness (30), enhance users' skills in using security tools (31), thereby improving user compliance behavior (UCB). Researchers suggest that for management, it is crucial to provide users with full commitment and support regarding best practices for laboratory safety behavior, as such exemplary leadership can influence users' awareness of laboratory safety.

### 2.4 Trust in organizations

Humaidi and Balakrishnan (17) demonstrated that trust in the organization's ISP can enhance employee compliance with the ISP, but there is currently no research on how trust in laboratory safety policies improves compliance. Trust has many definitions. From the psychological viewpoint, trust is defined as the willingness of an individual (a trustor) to accept vulnerability to the action of another individual (a trustee) (32), whereby an individual believes that he or she will not be taken advantage of by other individuals (33). Trust has also been defined as an individual's confidence in other people's honesty and belief (34). Yong Rhee (35) classified trust as a three-dimensional construct: (a) social trust, reflected in social culture; (b) organizational trust, defined as the degree of trustfulness of an organization (reflecting the working rules and norms of work activities in organizations); and (c) trust in others, the trust relationship between co-workers and employers. According to Tan and Lim (36), trust in an organization refers to an employee's willingness to be vulnerable to their organization's actions, and it has been shown to be a significant factor for motivating employee commitment and increasing organizational performance (37). This finding was supported by Utami et al. (38), who underscored the importance for an organization to gain the trust of users in order to increase their commitment to work. Shahnawaz and Goswami (39) also argued that the trust factor is important in building human relationships; in this case, the relationship between employee and employer. If users feel they are treated well by their employer, they will work harder in return to achieve organizational goals, and their commitments to the organization will also increase (37). Psychological security is the core element of laboratory safety management, while trust serves as the cornerstone for building psychological security (40). In the field of medical biology, professionals must demonstrate a high level of professional commitment when handling experimental data (41). However, due to insufficient understanding of safety concepts among some professionals, potential safety risks may arise. In such cases, trust in the work environment becomes particularly crucial, transcending mere adherence to safety policies (42). Given this, researchers argue that in medical biology laboratories, organizational trust should be embedded between users and management. This approach would enhance users' commitment to complying with safety protocols implemented in medical biology, thereby reducing the occurrence of safety incidents.

## 3 The current research

Although research has emphasized the importance of trust and laboratory safety, trust and user compliance with laboratory safety policies have received little attention. To ensure full adherence to organizational rules and policies in safeguarding the organization's assets and users, maintaining trust between users and the organization is crucial. In this study, TPB, PBC construction (SE; facilitation condition), and perceived trust (PT) were employed to develop a laboratory safety policy compliance (LSPC) model, aiming to provide a detailed theoretical framework for human behavior in laboratory safety policies within the context of medical biology laboratories in Zhejiang universities. TPB was modified to include SE and facilitation conditions [defined in this study as management support (MS)]. We focused on organizational factors such as the implementation of safety mechanisms and laboratory safety training, while monitoring and educating users on appropriate laboratory safety behaviors were considered an aspect of MS. Previous studies have explored the impact of knowledge, attitude, awareness, and communication on laboratory safety behaviors. However, to our knowledge, these studies have not yet evaluated the indirect influence of MS on the compliance behaviors of users of the Laboratory Safety Policies (LSP) toward laboratory safety policies through SE and PT. Although Zaidi (12) found no statistically significant correlation between safety compliance and knowledge among faculty laboratory users in public universities, this does not imply that safety knowledge is unimportant in practice. In fact, safety knowledge forms the foundation for understanding laboratory safety policies, while self-efficacy, confidence, and skills are key factors in translating this knowledge into practical actions. Therefore, even though knowledge itself may not directly predict compliance, it remains a crucial component in ensuring that users are both able and willing to adhere to safety regulations. In their 2023 study, Sheng et al. pointed out that laboratory safety incidents are closely associated with insufficient compliance of users with safety policies (1). Based on this, the present study aims to examine the factors influencing user compliance behavior (UCB), as well as the mediating roles of self-efficacy (SE) and perceived trust (PT) between management support (MS) and user compliance behavior (UCB), with a focus on the following specific research questions:

**RQ1**: How is management support (MS) associated with user compliance behavior (UCB) with laboratory safety policies (LSP) in biomedical university laboratories, and what is the relative contribution of self-efficacy (SE) and perceived trust (PT) in facilitating this relationship?
**RQ2**: In the medical biology laboratory of higher education institutions, do intervention factors mediate the relationship between management support (MS) and user compliance behavior (UCB) with laboratory safety policies (LSP)?

### 3.1 Distinctive nature of laboratory safety vs. information security

It is important to clarify why the theoretical model originally developed for information security policy compliance is not merely replicated but is particularly pertinent to laboratory safety. Information security primarily concerns the confidentiality, integrity and availability of digital assets; violations typically result in data breaches, financial loss or reputational damage. In contrast, laboratory safety—especially in medical biology laboratories—directly involves risks of physical injury, biological exposure, chemical burns, fire, and even fatality. Non–compliance can lead to immediate and irreversible harm to the user and to others. This fundamental difference has three implications for our model.

First, *perceived trust (PT)* becomes even more critical in laboratory settings. When personal safety is at stake, users rely heavily on the belief that management has provided effective protective equipment, standard operating procedures and emergency responses. Trust in the organization's commitment reduces the uncertainty inherent in high–risk environments, thereby promoting compliance. Second, *self–efficacy (SE)* in a laboratory context encompasses hands–on skills—such as correctly using biosafety cabinets, handling sharps, operating chemical fume hoods and performing emergency washing—rather than merely recognizing phishing emails or updating antivirus software. Third, *management support (MS)* in laboratories must go beyond documented policies; it requires visible leadership in safety drills, adequate provision of personal protective equipment, routine inspections and transparent reporting of incidents. These differences justify our expectation that the indirect effects of MS through both PT and SE are significant in the laboratory domain, and they also explain why PT may exhibit a stronger influence than SE, as users facing physical dangers may prioritize institutional trust over their own perceived competence. By explicitly adapting the TPB-based model to the distinctive features of biomedical laboratory work, this study provides a theoretically grounded and context–sensitive extension of prior compliance research.

### 3.2 Adaptation of the theory of planned behavior

While TPB includes attitudes and subjective norms as core predictors of intention, the present study focuses on perceived behavioral control (PBC) and trust. This choice is motivated by two considerations. First, in the context of laboratory safety compliance, managerial interventions (e.g., training, policy communication, and resource allocation) are more directly capable of shaping users' self-efficacy and perceived trust than altering general attitudes or subjective norms. Second, previous information security research that successfully applied TPB to compliance behavior also found that the PBC component—decomposed into self-efficacy and facilitating conditions (management support)—together with trust explained a substantial portion of variance in actual compliance, whereas attitudes and subjective norms contributed less in mandated organizational settings (17). Therefore, we adopt a parsimonious model that emphasizes facilitators of behavior control and trust, which are actionable levers for laboratory management.

## 4 Hypotheses development

### 4.1 MS, SE, and UCB

MS has been described as users' perception toward top management's commitment to protect information, one of the aspects in critical security components (43). Al-Salihy et al. (44) referred to MS as a commitment from the management in the organization as observed by users. Although the specific definition of management support is not widely available in the laboratory field, this study attempts to define it as users perception of senior management's commitment to safeguarding the laboratory. In any organization, the full support of management is critical to ensuring laboratory safety (45). In the field of laboratory management in higher education, management commitment typically includes developing and documenting the organization's laboratory safety policies and procedures, ensuring their effective implementation, and providing laboratory safety training and awareness programs (46). Empirical studies have demonstrated the impact of perceived management commitment on graduate students safety compliance and participation in laboratory settings. Research indicates that perceived management commitment positively influences both safety compliance and active engagement. This suggests that when graduate students perceive management's commitment to laboratory safety, they are more likely to adhere to safety protocols and actively participate in safety-related activities (47). In the current study, self-efficacy is described as the psychological process of an individual's confidence in their ability to perform a specific task or behavioral pattern (48). To enhance self-efficacy, comprehensive safety training must be provided to ensure researchers understand how to conduct experiments safely. Furthermore, active involvement and supervision by laboratory supervisors or managers can strengthen researchers' self-efficacy, as they motivate researchers to adopt safe behaviors by demonstrating and emphasizing the importance of safety (49). Therefore, management must educate users on the proper use of safety countermeasures and the rationale behind maintaining safe practices to effectively prevent laboratory safety threats. The application of user skills in implementing safety countermeasures is also recognized as a mediator of the impact of MS on compliance with laboratory safety policies. Consequently, we propose the following hypotheses:

**H1**: MS is positively associated with SE.
**H1(a)**: MS has an indirect association on user's compliance behavior toward LSP through SE.

The study also demonstrated that researchers with higher self-efficacy were more likely to comply with laboratory safety policies, such as the proper use of personal protective equipment (PPE) (49). Self-efficacy is a significant determinant of patient participation in promoting self safety and preventing medical errors. Patients with higher self-efficacy are more likely to adopt safety behaviors, such as asking healthcare providers to wash their hands before procedures and seeking second opinions when necessary (50). We therefore propose the following hypothesis:

**H2**: SE is positively associated with users' compliance behavior toward LSP.

### 4.2 MS, PT, and UCB

PT refers to users' expected confidence on the implementation of safety policies through managerial support. The perception of trust among users depends on the subjective needs of the users and social constraints. If users have higher trust in the security system, they will likely use the security system consistently, which, in turn, may reduce security incidents in the organization (51). Lippert and Davis (52) also support this view, arguing that trust is a critical factor in organizations as it influences users' willingness to adopt implemented safety technologies. Given this perspective, it is believed that in the field of medical biology, trust should be established between users and laboratory management. Consequently, users will be more committed to adhering to laboratory safety policies implemented in medical biology, thereby reducing safety incidents. Therefore, we formulate the following hypothesis:

**H3**: MS is positively associated with PT.
**H3(a)**: MS has an indirect association on users' compliance behavior toward LSP through PT.
**H4**: PT is positively associated with users' compliance behavior toward LSP.
**H5**: Management support is positively associated with users' compliance behavior toward laboratory safety policies.

## 5 Method

### 5.1 Design of the study

This study employs a questionnaire survey design. A convenience sampling approach was adopted, as a complete sampling frame of all laboratory users across institutions was not available. Within each selected institution, target sample sizes were set for three strata (undergraduate students, graduate students, and in-service researchers) proportional to their estimated representation in the population. This approach is best described as convenience sampling with stratified allocation targets. Self-administered questionnaires were distributed to selected laboratory users. The questionnaire, constructed based on TPB, includes assessments of MS, SE, PT, and UCB. Researchers reviewed previous literature to assist in the development of the LSPC model and to test research hypotheses. In Zhejiang Province, China, university biomedical laboratories are categorized into three types: biomedical laboratories in medical universities, biomedical laboratories in medical schools under secondary management of comprehensive universities, and biomedical laboratories in universities without medical schools. This study focuses solely on the first two types of laboratories, as they are more comprehensive. Before the study commenced, we arranged an internal university meeting and invited the Principal Investigator (PI), experimental management users, and college management users to participate in the meeting to further clarify the rationale and current status of implementing LSR in the selected university. Before commencing data collection, we submitted the necessary approval application to the university's ethics committee.

### 5.2 The research model

The LSPC model utilized in this study comprises three exogenous constructs (independent variables): MS, SE, and PT. The MS construct is assessed through leadership behavior and the implementation of LSP training and education, whereas SE and PT are included as mediators. The endogenous construct (dependent variable) of this study is the UCB pertaining to laboratory safety Policies (LSP), as illustrated in Figure 1.

**Figure 1:**
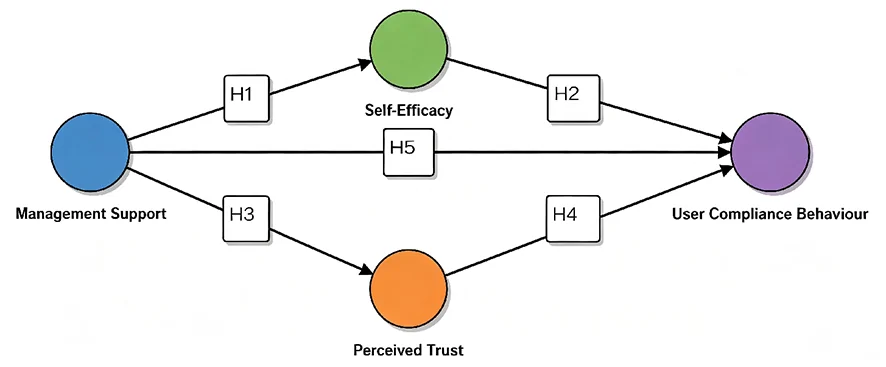
LSPC model. LSPC, laboratory safety policies compliance.

### 5.3 Sampling procedure and participants

Once approved by the university's ethics committee, the questionnaire was distributed to selected respondents in university laboratories, and quantitative data were collected through self-administered surveys.

The selection of institutions and laboratories was based on accessibility to the research team. Within each selected laboratory, all eligible users were invited to participate voluntarily via the Questionnaire Star platform. Eligibility was defined as individuals conducting research or practical work in biomedical laboratories at the selected universities.

To ensure adequate representation of different user groups, we set target sample sizes for three strata—undergraduate students, graduate students, and in-service researchers—proportional to their estimated distribution in the target population. The total estimated population was 894, comprising 840 undergraduates, 22 graduate students, and 32 in-service researchers. However, because no complete sampling frame was available, the allocation targets were applied as guidance rather than as strict probability-based quotas.

Graduate students and in-service researchers, though small in absolute numbers, were nearly fully covered within the selected institutions, as their total population in the target laboratories was limited. The undergraduate sample, by contrast, represents a larger but proportionally smaller fraction of the undergraduate population, reflecting their predominance in teaching laboratories.

A pilot test (*n* = 50) was conducted prior to the main survey; these respondents were excluded from the final sample. Participation was voluntary, and no incentives were provided. All participants provided informed consent. Between December 2024 and April 2025, we distributed 520 questionnaires; 510 were valid and included in the analysis, exceeding the minimum sample size required for the PLS-SEM analysis.

### 5.4 Survey instrument

The questionnaire is divided into three sections: Section A includes demographic questions such as sex, age, role, and years of experience in the laboratory. Section B assesses the perceptions of laboratory researchers toward trust related to MS, SE, and LSP. Section C evaluates the attitudes of laboratory researchers toward UCB's laboratory safety policies. The questionnaire is adapted from Humaidi and Balakrishnan (17). All indicators in Sections B and C are measured using a Likert 7-point scale, with anchor points ranging from 1 (strongly disagree) to 7 (strongly agree). A brief description of individual survey items is provided in **Appendix 1**. Before conducting the survey, the content validity of the questionnaire was evaluated. The survey instrument was adapted from Humaidi and Balakrishnan (17) through a four-stage localization process. First, the original English items were translated into Mandarin Chinese by a bilingual expert in laboratory safety management. Second, an independent translator performed back-translation into English; discrepancies between the original and back-translated versions were resolved through discussion. Third, a panel of five content experts (three laboratory safety managers and two behavioral researchers) evaluated item relevance using a four-point scale; the resulting Content Validity Index (CVI) exceeded 0.80 for all items. Fourth, a pilot test with 50 respondents (excluded from the main sample) confirmed satisfactory internal consistency (Cronbach's α ranging from 0.713 to 0.917), and three items with inter-item correlations below 0.30 were revised before the main survey. All 17 management support items were treated as a single construct because they collectively reflect the broader theoretical concept of perceived management commitment to laboratory safety. In line with prior research on management support in safety contexts (43, 47), these items capture various manifestations of the same underlying organizational phenomenon: users' perceptions of whether management prioritizes, communicates, and enforces safety policies. Although the items cover multiple operational aspects (leadership, training, resources, and discipline), they are all indicators of the same higher-order construct—perceived management support—rather than distinct dimensions. Empirically, the high factor loadings (0.834–0.925) and high composite reliability (CR = 0.984) support the internal consistency of treating them as a single construct.

### 5.5 Data analysis

Partial least squares structural equation modeling (PLS-SEM) was applied using SmartPLS version 4.0 (53) to test the measurement model and the structural model. PLS-SEM was chosen because it is prediction-oriented and does not require normally distributed data (54).

The PLS-SEM analysis was conducted with the following settings: the path weighting scheme was used for model estimation, and indicators were specified as reflective. Bootstrapping with 5,000 sub-samples was applied to estimate standard errors and construct 95% bias-corrected and accelerated (BCa) confidence intervals. Missing data were handled by listwise deletion (less than 1% of responses were missing). No extreme outliers were detected based on visual inspection of the data. Predictive relevance was assessed using the Stone–Geisser Q^2^ criterion via the blindfolding procedure.

Predictive relevance of the structural model was assessed using the Stone–Geisser Q^2^ criterion via the blindfolding procedure with an omission distance of 7.

### 5.6 Common method bias assessment

Although procedural remedies such as anonymous questionnaires were implemented, we conducted *post-hoc* statistical tests to assess the potential threat of common method bias (CMB) (55). First, Harman's single-factor test was performed using principal component analysis on all 29 measurement items. The first unrotated factor accounted for 64.17% of the total variance, exceeding the recommended threshold of 50%. Second, we applied Kock's full collinearity test (56) in SmartPLS. The variance inflation factor (VIF) values for all indicators ranged from 3.57 to 7.00, with the majority falling between 5 and 6, all above the conservative threshold of 3.3.

These results suggest that common method bias may be present in the data. However, it is important to note that CMB inflates rather than creates statistically significant relationships (57), and the magnitude of this bias in the context of highly correlated theoretical constructs (e.g., management support, trust, and compliance) remains debated. Nevertheless, we acknowledge this limitation and interpret our findings with caution. The implications of CMB are further addressed in the Limitations section.

## 6 Results

### 6.1 Descriptive data

Demographic data relating to the participants in this study are outlined in Table 1. Table 1 shows the number of undergraduate, Master's student, PhD student and research users member and Teacher across the selected university who participated in the study, while Table 1 provides the demographic profile of all study participants.

**Table 1:** Demographical profile of study participants.

Demographics	n = 510	
	Frequency	Percentage
Sex		
Male	165	32.35
Female	345	67.65
Age		
18–25	468	91.76
26–35	24	4.71
36–45	13	2.55
>45	5	0.98
Role		
Undergraduate	478	93.72
Graduate students	14	2.75
In-service researchers	18	3.53
Years of experience in laboratory		
<1year	369	72.35
1–3 years	119	23.33
4–5 years	10	1.96
>5 years	12	2.36

### 6.2 Measurement model

Confirmatory factor analysis results (Table 2) showed that all indicators measuring a particular construct had loading values of more than 0.7 on their respective constructs. Specifically, the factor loadings ranged from 0.712 to 0.925, exceeding the recommended threshold of 0.7 (58). These results confirmed that all indicators were highly valid for their respective constructs and demonstrated strong indicator reliability. As suggested by Hair (59), we examined composite reliability (CR) and average variance extracted (AVE) to assess the convergence validity, as shown in Table 3. The CRs for each construct ranged from 0.954 to 0.984, which exceeded the recommended value of 0.7. The AVE for each construct ranged from 0.794 to 0.909, which is greater than 0.5. Thus, the cut-off values ensured that at least 50% or more of the variances in the constructs could be explained by the set of indicators. The collected data had previously been verified for its reliability by calculating Cronbach's alpha. The resulting values ranged from 0.954 to 0.984, which was deemed acceptable. Results of the measurement model showed that all the four constructs were valid measures based on their parameter estimates and statistical significance (58).

**Table 2:** Factor loadings and cross loadings.

Constructs/items	MS	PT	SE	UCB
MS01	**0.842**	0.695	0.653	0.657
MS02	**0.849**	0.666	0.671	0.634
MS03	**0.878**	0.710	0.681	0.683
MS04	**0.894**	0.686	0.688	0.651
MS05	**0.875**	0.716	0.704	0.699
MS06	**0.901**	0.701	0.717	0.676
MS07	**0.906**	0.734	0.716	0.709
MS08	**0.898**	0.713	0.722	0.685
MS09	**0.913**	0.735	0.726	0.697
MS10	**0.918**	0.731	0.738	0.712
MS11	**0.925**	0.745	0.744	0.704
MS12	**0.888**	0.727	0.719	0.717
MS13	**0.924**	0.766	0.741	0.746
MS14	**0.907**	0.753	0.741	0.719
MS15	**0.834**	0.659	0.670	0.627
MS16	**0.897**	0.737	0.734	0.698
MS17	**0.891**	0.768	0.714	0.740
PT01	0.768	**0.951**	0.753	0.767
PT02	0.778	**0.935**	0.771	0.736
PT03	0.758	**0.947**	0.743	0.766
PT04	0.756	**0.948**	0.739	0.784
SE01	0.758	0.758	**0.941**	0.745
SE02	0.757	0.730	**0.933**	0.719
SE03	0.739	0.739	**0.939**	0.701
SE04	0.738	0.752	**0.936**	0.726
UCB01	0.735	0.770	0.732	**0.956**
UCB02	0.742	0.781	0.737	**0.955**
UCB03	0.742	0.754	0.733	**0.944**
UCB04	0.744	0.774	0.740	**0.958**

MS, management support; PT, perceived trust; SE, self-efficacy; UCB, user compliance behavior. Bold values indicate factor loadings of items on their own construct; non-bold values represent cross-loadings.

**Table 3:** Convergent validity, reliability, and discriminant validity.

Construct	AVE	CR	*R* ^2^	α	MS	PT	SE	UCB
MS	0.794	0.984		0.984	**0.891**			
PT	0.894	0.960	0.655	0.960	0.809	**0.945**		
SE	0.878	0.954	0.637	0.954	0.798	0.795	**0.937**	
UCB	0.909	0.967	0.698	0.966	0.777	0.807	0.771	**0.953**

Values in bold are the square roots of AVE (Fornell-Larcker criterion). Off-diagonal values are latent-variable correlations. AVE, average variance extracted; CR, composite reliability; α, Cronbach's alpha; MS, management support; PT, perceived trust; SE, self-efficacy; UCB, user compliance behavior.

Furthermore, we assessed the heterotrait-monotrait ratio (HTMT). Henseler et al. (60) suggested a threshold of 0.85; all HTMT point estimates were below this criterion (the highest value was 0.838 between PT and UCB). However, bootstrap 95% confidence intervals (reported in parentheses in Table 4) revealed that the upper bounds for several construct pairs (e.g., PT–UCB: 0.928) exceeded 0.85, indicating some uncertainty regarding discriminant validity. This suggests that perceived trust and compliance behavior are closely related but conceptually distinguishable constructs. Readers should interpret the PT–UCB relationship with caution, and future research may benefit from additional items that better differentiate trust from behavioral compliance.

**Table 4:** HTMT ratios (below diagonal) and 95% bootstrap confidence intervals (upper bounds in parentheses).

	MS	PT	SE	UCB
MS	–			
PT	0.832 (0.915)	–		
SE	0.824 (0.904)	0.831 (0.913)	–	
UCB	0.796 (0.880)	0.838 (0.928)	0.803 (0.888)	–

As part of the full collinearity assessment for CMB, we examined the variance inflation factor (VIF) for all indicators. The VIF values ranged from 3.57 to 7.00, with a mean of 5.50. Detailed VIF values are available upon request. These values exceed the common threshold of 3.3 (56), further indicating potential CMB.

### 6.3 Structural model

Before examining the structural paths, we assessed overall model fit using the standardized root mean square residual (SRMR). The SRMR for the estimated model (including the direct path MS → UCB) was 0.038, which is below the recommended threshold of 0.08 (60), indicating acceptable model fit. The normed fit index (NFI) was 0.915, further supporting the adequacy of the model specification.

Predictive relevance was assessed using the Stone–Geisser Q^2^ criterion via the blindfolding procedure. The Q^2^ values for the endogenous constructs were: Perceived Trust (Q^2^ = 0.580), Self-Efficacy (Q^2^ = 0.555), and User Compliance Behavior (Q^2^ = 0.645). All Q^2^ values were greater than zero, indicating adequate predictive relevance of the structural model (58).

Results showed that 63.7% of variance in SE and 65.5% of variance in PT were explained by the MS and that 69.8% of variance in UCB of LSR safety policies was explained by SE and PT (see Figure 2). Results also showed that MS has the strongest association with PT (β =0.809, *t*-value = 18.478), followed by SE (β = 0.798, *t*-value = 18.552). PT was significantly associated with UCB toward LSP related to LSR uses (β = 0.411, *t*-value = 3.956). Direct effect of SE on UCB toward LSP was also significant (β = 0.248, *t*-value = 3.265). Therefore, all hypotheses were supported, as shown in Table 5, and RQ1 was answered. Bootstrapping analysis showed that the indirect effect of MS through SE (β = 0.198) and PT (β = 0.332) was significant, with *t*-values of 3.095 and 3.522, respectively. As suggested by (61), the indirect effects 95% boot confidence interval [(SE: lower limit=0.082, upper limit = 0.335) and (PT: lower limit = 0.190, upper limit = 0.564)] did not straddle a 0 in between, indicating there was a mediation effect. Thus, RQ2 was answered and we were able to conclude that the mediation effects were statistically significant, as shown in Table 5.

**Figure 2:**
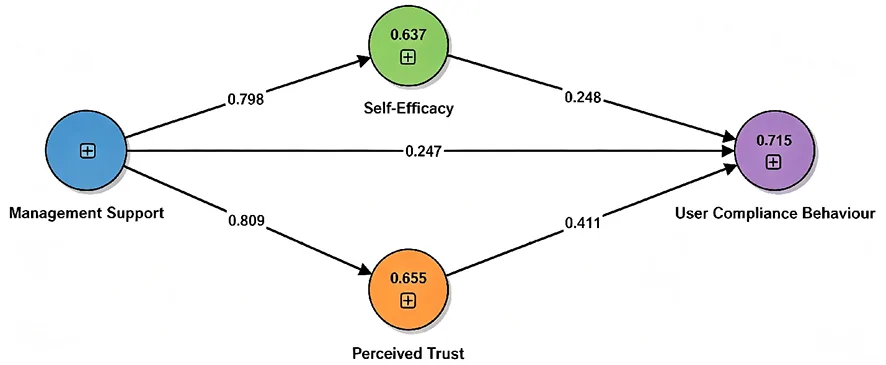
LSPC model—standard path results. LSPC, laboratory safety policies compliance.

**Table 5:** Hypothesis testing.

Hypotheses	Relationship	β	Standard error	*t*-values	*P* values	*f* ^2^
H1	MS → SE	0.798	0.043	18.552	<0.001	1.756
H2	SE → UCB	0.248	0.076	3.265	0.001	0.064
H3	MS → PT	0.809	0.044	18.478	<0.001	1.897
H4	PT → UCB	0.411	0.104	3.956	<0.001	0.168
H5	MS → UCB	0.247	0.084	2.948	0.003	0.060
H1(a)	MS → SE → UCB	0.198	0.064	3.095	0.002	–
H3(a)	MS → PT → UCB	0.332	0.094	3.522	<0.001	–

MS, management support; SE, self-efficacy; UCB, user compliance behavior; PT, perceived trust.

To assess whether the difference between the two indirect effects was statistically significant, we computed the difference between the indirect effect via PT and via SE for each of the 5,000 bootstrap samples. The 95% bootstrap confidence interval for the difference (PT minus SE) was [–0.117, 0.465], which includes zero. This indicates that the two indirect effects were not significantly different from each other, despite the numerical difference in their point estimates (PT: β = 0.332; SE: β = 0.198). Thus, the apparent stronger indirect effect through perceived trust should be interpreted with caution.

## 7 Discussion

This study introduced a Laboratory Safety Policy Compliance (LSPC) human behavior model by positing the mediating effects of perceived trust (PT) and self-efficacy (SE) within the context of laboratory safety management in higher education institutions. This particular combination of behavioral constructs has not received much attention from scholars investigating laboratory safety compliance among university researchers, particularly in the context of medical and biological laboratories in China. The study also addresses a research gap by examining the relationship between different dimensions of management support (MS) and compliance behavior, extending the application of the theory of planned behavior (TPB) to the domain of laboratory safety. The analysis revealed that MS was significantly and substantially associated on both SE and PT among laboratory users. In particular, MS showed the strongest association on PT (β = 0.809), followed by SE (β = 0.798). These findings suggest that when laboratory users perceive strong commitment from management—through clear safety policies, regular training, and visible leadership—they are more likely to develop confidence in the organization's safety system and in their own ability to perform safe behaviors. This is consistent with previous studies in information security (17) and laboratory safety (47), which emphasized the critical role of top management in shaping employee behavior. The effect of PT on user compliance behavior (UCB) toward laboratory safety policies (β = 0.411) was numerically larger than that of SE (β = 0.248). However, a formal bootstrap test of the difference between the two indirect effects indicated that this difference was not statistically significant (95% CI [–0.117, 0.465]). Thus, while perceived trust appeared descriptively more strongly associated with compliance behavior than self-efficacy, both mediators played significant and complementary roles. This pattern aligns with prior research highlighting the importance of both institutional trust and personal competence in safety compliance (40, 42), which highlighted that psychological safety and organizational trust are foundational for sustained compliance, particularly in high-risk environments such as medical biology laboratories. In such settings, where experimental complexity and potential hazards are high, users may rely heavily on their trust in institutional safeguards to guide their behavior.

The numerically larger association of PT (compared to SE) with compliance behavior may be attributed to the distinctive characteristics of biomedical laboratory environments. First, when non-compliance can lead to immediate physical harm (e.g., chemical burns, biological exposure, or fire), users may rely heavily on institutional assurance. Second, the majority of our participants were inexperienced undergraduates (see Limitations), who often lack sufficient hands-on training and thus may rely on trust in management's commitment as a heuristic for safe behavior. In contrast, self-efficacy requires accumulated mastery experiences, which take time to develop. Consequently, in teaching-oriented universities where inexperienced users dominate, fostering perceived trust may be a more immediately effective leverage point than enhancing self-efficacy alone. Nevertheless, given the non-significant difference between the two indirect pathways, both mechanisms should be considered in intervention design. The mediation analyses further demonstrated that MS indirectly influenced UCB through both SE and PT, with the indirect effect through PT (β = 0.332) being numerically larger than that through SE (β = 0.198). However, a formal comparison of the two indirect effects indicated that this difference did not reach statistical significance (95% CI [–0.117, 0.465]). These results underscore the importance of fostering both trust and self-efficacy as complementary mechanisms through which management support is associated with compliance behavior. This finding is particularly relevant for university laboratories, where users—especially undergraduate and graduate students—may have limited experience and rely on institutional signals of safety commitment. Thus, laboratory management should not only focus on providing training and resources but also on building a transparent, fair, and trustworthy safety culture.

### 7.1 Direct effect of management support on compliance behavior

In addition to the indirect pathways, management support also exerted a significant direct effect on user compliance behavior (H5: β = 0.247, *p* = 0.003). This indicates that management support not only enhances compliance by fostering trust and self-efficacy but also directly encourages compliance through mechanisms such as visible leadership, consistent rule enforcement, and immediate feedback (62). The direct effect is modest compared to the total indirect effect (β_total indirect_ = 0.530), suggesting that the primary influence of management support operates through psychological mediators (trust and self-efficacy). The presence of partial mediation (rather than full mediation) is theoretically plausible: in the context of laboratory safety, management actions such as routine safety inspections and explicit disciplinary consequences may directly shape behavior without necessarily altering users' internal psychological states. This pattern aligns with prior safety research in organizational settings (63).

### 7.2 Limitation of common method bias

A major limitation of this study is the potential influence of common method bias (CMB). All variables were measured via self-report at a single time point, and *post-hoc* diagnostic tests indicated a substantial risk: Harman's single-factor test explained 64.17% of the variance, and full-collinearity VIF values ranged from 3.57 to 7.00. Consequently, the reported path coefficients, particularly the mediation effects, may be inflated. While the comparison between the two indirect effects (PT vs. SE) remained consistent in direction, the absolute magnitude of these coefficients should be interpreted with caution. We therefore present CMB as a central limitation of this study, rather than a routine methodological caveat. Future research should consider multi-source, time-lagged designs to reduce the risk of CMB.

Taken together, the findings suggest that in the context of Chinese university biomedical laboratories, management support functions not only as a direct driver of safety behavior but also as a critical enabler of psychological states—namely self-efficacy and perceived trust—that mediate the path to compliance. These results extend the application of TPB by demonstrating that facilitating conditions (operationalised as MS) influence behavioral outcomes through internal cognitive and affective mechanisms, rather than solely through direct effects on behavioral intention.

## 8 Limitations

The study has several limitations that should be acknowledged. First, the sample was drawn exclusively from university biomedical laboratories in Zhejiang Province, China, which may limit the generalizability of the findings to other types of laboratories (e.g., chemical, engineering, or physical sciences) or to other institutional contexts such as industrial or government research settings. Future studies could extend the sample to include a broader range of laboratory types and geographic regions to enhance the external validity of the model.

Second, the majority of participants were undergraduate students (93.72%), with relatively limited laboratory experience (72.35% had less than one year of experience). While this reflects the actual demographic composition in teaching–oriented universities where undergraduates are the primary laboratory users, it may limit the generalizability of our findings to research–intensive universities or to professional research settings (e.g., postdoctoral fellows, principal investigators, full–time technicians). In teaching–oriented institutions, undergraduate students often follow structured protocols with close supervision, whereas experienced researchers in research–focused environments may exhibit different compliance patterns due to greater autonomy and deeper safety training. Therefore, future research should compare compliance behavior across different types of higher education institutions (teaching vs. research–oriented) and across user experience levels to examine whether the mediating roles of self–efficacy and perceived trust remain consistent.

Third, and most importantly, this study is subject to a substantial risk of common method bias (CMB), as all data were collected via self-reported questionnaires at a single time point. *Post-hoc* statistical tests (Harman's single-factor test: 64.17% variance explained by the first factor; full collinearity VIF values mostly between 5 and 6) indicated that CMB may be present. Consequently, the observed relationships, particularly the path coefficients, could be inflated. We have therefore interpreted the results with caution, focusing on the pattern of significance and the relative strength of indirect effects (PT vs. SE) rather than on absolute effect sizes. We emphasize that this is a central limitation of the present study. Readers should interpret the reported coefficients as associations that may be inflated by shared method variance, rather than as precise estimates of true effect sizes.

To mitigate CMB in future research, several design improvements are recommended: (a) collecting predictor and criterion variables at different time points; (b) obtaining data from multiple sources (e.g., combining self-reported perceptions with supervisor-rated compliance or objective safety records); (c) incorporating a theoretically unrelated marker variable in the questionnaire to allow statistical control; and (d) using reverse-coded items or different scale anchors to reduce response consistency motives.

Fourth, the cross-sectional design of the study limits the ability to draw causal inferences. Although the structural model was specified based on strong theoretical foundations, the relationships observed are correlational in nature. Longitudinal studies would be valuable to establish temporal precedence and to examine how changes in management support over time influence self-efficacy, trust, and compliance behavior.

Finally, the study focused on a specific set of constructs derived from TPB and prior information security literature. Other potentially important factors—such as safety culture, peer influence, risk perception, or physical work environment—were not included in the model. Future research could integrate these additional factors to develop a more comprehensive understanding of laboratory safety compliance behavior.

## 9 Conclusions

This study contributes to the growing body of research on laboratory safety by developing and empirically testing a Laboratory Safety Policy Compliance (LSPC) model based on the theory of planned behavior, extended with constructs of self-efficacy and perceived trust as mediators. The model was tested using partial least squares structural equation modeling (PLS-SEM) with data collected from 510 laboratory users in university biomedical laboratories in Zhejiang Province, China.

The results reveal that management support plays a crucial role in shaping both self-efficacy and perceived trust among laboratory users. Furthermore, perceived trust was found to be the strongest predictor of user compliance behavior, highlighting the importance of building a trustworthy safety environment. Both self-efficacy and perceived trust served as significant mediators in the relationship between management support and compliance behavior, with trust mediating a larger proportion of the indirect effect.

These findings offer several practical implications for university laboratory management, particularly within the framework of China's Ministry of Education Laboratory Safety Policy (2023). According to Article 6 of the Ministry's guidelines, higher education institutions are required to “establish and improve a project risk assessment and management and control mechanism, rely on modern technical means to strengthen information technology construction, and build a full-cycle laboratory safety management mechanism” (46). To translate these official requirements into actionable management practices and to address the common implementation gaps observed in many universities, we propose the following eight specific measures [The following recommendations combine three types of evidence: (a) those directly supported by the empirical findings of this study (items 1, 2, 5, and 7); (b) those derived from national policy documents (items 3, 4, and 8); and (c) broader expert proposals informed by the theoretical framework (item 6).]:

(1) Institutionalize a transparent near-miss reporting system that rewards voluntary disclosure of safety hazards and close calls rather than penalizing reporters, thereby building perceived organizational fairness—a core component of trust.
(2) Embed laboratory safety compliance metrics into the annual performance evaluation of Principal Investigators (PIs), department heads, and laboratory supervisors, establishing clear accountability chains.
(3) Digitize the full safety management cycle using modern information technologies (e.g., barcode-based chemical tracking, electronic safety passports, and real-time hazardous condition dashboards) to enable traceability and early warning, directly following the Ministry's call for digital transformation.
(4) Link each experimental protocol to a corresponding risk rating and mandated safety training module, ensuring that no experiment proceeds without both a risk clearance certificate and a user's verified competency record.
(5) Require PIs to conduct monthly safety briefings that explicitly connect institutional safety policies to the specific experimental workflows of undergraduate users, reinforcing behavioral relevance.
(6) Establish a routine cross-departmental safety inspection system with standardized checklists and closed-loop follow-up mechanisms (inspection → feedback → rectification → verification).
(7) Launch a mandatory safety competency certification programme that must be renewed annually, covering chemical handling, emergency response, biosafety protocols, and equipment operation.
(8) Integrate laboratory safety governance into the university's institutional risk management framework by creating a centralized office or committee with dedicated funding and enforcement authority, moving beyond fragmented departmental efforts to a systematic governance structure.

These eight measures collectively address the root cause of policy ineffectiveness identified in recent institutional audits: policies are often well designed on paper but poorly enforced in practice. By shifting the focus from policy proclamation to systematic execution—through digitisation, accountability embedding, and enforcement infrastructure—laboratory safety management can move beyond symbolic compliance toward genuine behavioral change.

Readers should be aware that the presence of common method bias may have inflated the reported effect sizes. Nevertheless, both indirect pathways (via PT and via SE) remained significant across ancillary analyses, suggesting that the mediation effects are unlikely to be solely artifacts of CMB. The numerical difference between the two pathways, while not statistically significant, points to the potential importance of trust-building as a complementary strategy alongside efficacy-enhancing interventions.

In conclusion, ensuring laboratory safety is not solely a matter of enforcing rules; it requires cultivating an environment in which users feel capable, supported, and trusting of the systems designed to protect them. As universities continue to expand their research capacity, investing in management practices that build trust and self-efficacy will be essential for sustaining safe and productive laboratory environments.

## Data Availability

The original contributions presented in the study are included in the article/supplementary material, further inquiries can be directed to the corresponding author.

## Appendix 1

Descriptive analysis of each items used to measure independent variables and dependent variable.

**Table A1:** Independent variables.

**MS: leadership behavior**
MS01: The management often publicly emphasizes the importance of laboratory safety.
MS02: The management has implemented clear penalties for violations of laboratory safety policies.
MS03: The management has provided sufficient resources to support the implementation of safety measures in the laboratory.
**MS: Laboratory safety training and policy implementation**
MS04: The management team always provides specialized training on laboratory safety on a regular basis.
MS05: My supervisor encouraged me to participate in any laboratory safety training.
MS06: The laboratory safety training organized by the management has been completed.
MS07: The laboratory safety training organized by the management is effective.
MS08: The management team has efficiently documented the laboratory safety policies, making it easy for me to understand them.
MS09: In my organization, the laboratory safety policy is easily accessible.
MS10: The management team informed me of the changes related to the laboratory safety policy.
MS11: In the event of non-compliance with the organizational laboratory safety policy, there is a clearly defined disciplinary structure.
MS12: The leader believes that if I adopt appropriate laboratory safety behaviors, my work performance will improve.
MS13: The safety policy of my organisation's laboratory helps me understand how to take appropriate actions when dealing with matters related to laboratory safety.
MS14: Laboratory safety articles or current affairs notifications will be distributed to all users in my organization.
MS15: All users in my organization receive alerts regarding laboratory safety threats via text message, email, or other communication methods.
MS16: All users in my organization are reminded of the laboratory safety policies at any time through text messages, emails, or other communication methods.
MS17: The management users organize continuous laboratory safety activities to enhance the safety awareness of laboratory users.
**Self-efficacy**
SE01: I am capable of identifying and promptly addressing potential safety hazards in the laboratory.
SE02: I understand how to properly handle hazardous chemicals in the laboratory to prevent safety accidents.
SE03: I am capable of independently operating laboratory safety equipment and taking appropriate measures in emergency situations.
SE04: When confronted with safety issues that have arisen in the laboratory, I am capable of effectively implementing countermeasures.
**Perceived trust**
PT01: I believe that our organization has taken into account the safety and well-being of teachers and students in all of its decisions.
PT02: I am satisfied with the transparency and fairness of the organization in handling security issues.
PT03: I believe the organization will take necessary measures to protect teachers and students from the risks associated with laboratory safety.
PT04: I believe in the organisation's commitment to laboratory safety and trust that these policies are designed to protect everyone's safety.
**Dependent variable**
**Laboratory safety policies compliance behavior**
UCB01: I always adhere to all safety regulations in the laboratory, even when unsupervised.
UCB02: I actively seek to improve my laboratory safety practices to mitigate any potential risks.
UCB03: I often discuss laboratory safety issues with my classmates and encourage them to adhere to safety regulations.
UCB04: I actively promote and implement safety measures in the laboratory to ensure the safety of all users.

MS, management support; SE, self-efficacy; UCB, user compliance behavior.
